# Official Privileges Extended to Women Physicians

**Published:** 1898-10

**Authors:** 


### Official Privileges Extended to Women Physicians.

A recent decree places women occupying official medical positions in Russia on the same footing as men, with the exception of promotions, wearing uniforms and decorations. They do not lose their right to a pension if they marry, and their orphan children inherit it. The decree only refers to a limited number who completed their medical course under certain circumstances. Woman has long been legally on an equality with man in Russia.

